# Supplementary material for: The genome sequence of Carex extensa Gooden., 1794 (Poales: Cyperaceae)
Source: Wellcome Open Res. 2026 Aug 6;11:511. [Version 1] doi: 10.12688/wellcomeopenres.27281.1 (PMC13752446; doi:10.12688/wellcomeopenres.27281.1)
Supplement: Peer review history

# Reviewer report: Reviewer response for version 1

**DOI:** 10.21956/wellcomeopenres.30025.r175930

**Published:** 2026-11-10

**Authors:** Steven Newmaster

**Affiliations:** 1 University of Guelph, Guelph, Ontario, Canada

I have reviewed the manuscript entitled “The genome sequence of Carex extensa Gooden., 1794 (Poales: Cyperaceae)”.  The methods are appropriate and well-developed and verified through considerable research by the Darwin Tree of Life project group of researchers, who have already indexed reference genomes for many plant species. These methods provide a high-quality genome assembly estimate for this sample that is annotated ready and for indexing and use by other research scientist working on Carex and related species. This is also an important indexing given the ecological habitat of this species in coastal wetlands. One addition I suggest is to add the location of where the voucher specimens were archived including herbarium accession numbers.

Are sufficient details of methods and materials provided to allow replication by others?

Yes

Is the rationale for creating the dataset(s) clearly described?

Yes

Are the datasets clearly presented in a useable and accessible format?

Yes

Are the protocols appropriate and is the work technically sound?

Yes

Reviewer Expertise:

botany, taxonomy, systematics, genomics, floristics

I confirm that I have read this submission and believe that I have an appropriate level of expertise to confirm that it is of an acceptable scientific standard.

# Reviewer report: Reviewer response for version 1

**DOI:** 10.21956/wellcomeopenres.30025.r167445

**Published:** 2026-09-11

**Authors:** Samuel J. Smit

**Affiliations:** 1 University of York, York, England, UK

The authors provide a high-quality phased assembly of *Carex extensa. *This ecologically important Angiosperm provides a valuable resource for studying the genetic drivers behind adaptation to growth in saline environments. The methods are clearly described, assembly is complete and the data is accessible. Haplotype 1 is at chromosome level and annotated. This genomic resource is well prepared and ready for public use.

Are sufficient details of methods and materials provided to allow replication by others?

Yes

Is the rationale for creating the dataset(s) clearly described?

Yes

Are the datasets clearly presented in a useable and accessible format?

Yes

Are the protocols appropriate and is the work technically sound?

Yes

Reviewer Expertise:

Plant genome assembly and comparative genomics.

I confirm that I have read this submission and believe that I have an appropriate level of expertise to confirm that it is of an acceptable scientific standard.
